# Optimizing Health and Athletic Performance for Women

**DOI:** 10.1007/s12178-021-09735-2

**Published:** 2022-01-13

**Authors:** Celina de Borja, Cindy J. Chang, Rhonda Watkins, Carlin Senter

**Affiliations:** 1 Department of Orthopaedic Surgery, Division of Pediatric Orthopaedics, University of California, San Francisco, 1825 4th Street, 5th Floor, San Francisco, CA 94158 USA; 2 Department of Orthopaedic Surgery, Primary Care Sports Medicine, University of California, San Francisco, San Francisco, CA 94158 USA

**Keywords:** Female athlete, Team physician, Women in sports medicine

## Abstract

**Purpose of Review:**

The exponential growth of women participating in competitive sports throughout the years was made possible through several initiatives by the International Olympic Committee and the passage and implementation of Title IX as a federal law in the United States. However, this positive trend towards gender equity in sports has not transpired for women in medicine, especially in fields that care for elite athletes. This current review will discuss specific areas that can be tailored to help female athletes prevent injuries and optimize their athletic performance. We will also highlight how increased female team physician representation in sports may help optimize care for female athletes.

**Recent Findings:**

Female athletes are considered high risk for certain conditions such as ACL tears, patellofemoral pain syndrome, bone stress injuries, sport-related concussions, and sexual violence in sport. Addressing factors specific to female athletes has been found to be valuable in preventing injuries. Strength and conditioning can optimize athletic performance but remains underutilized among female athletes. Although diversity in healthcare workforce has been found to be beneficial for multiple reasons, women remain underrepresented in sports medicine. Increasing female team physician representation may positively impact care for female athletes.

**Summary:**

Team physicians must understand the physiologic, biomechanical, and anatomic factors that are unique to female athletes in order to tailor injury prevention programs and optimize their athletic performance. Advocating for gender equity in sports medicine to advance representation of women in the field will increase workforce diversity and promote excellence in sports medicine care.

## Introduction

During the 2016 Olympics in Rio de Janeiro, a new record was reached when 45% of the 11,444 athletes that competed were women [1]. Organizers of the first modern Olympics held in 1896 did not allow women to participate. Back then, women’s participation in competitive sport was at odds with the prevailing beliefs that women who were gentle, passive, and frail were more beautiful and more desirable. Misconceptions regarding physical exertion as a threat to a woman’s reproductive capabilities also misled the public to believe that women should only participate in sports considered to be feminine and leisurely [2,3]. It was not until 1900 that women were allowed to compete in a global arena for the first time [1]. Gradually, female athletes have debunked myths and broken barriers, shown through the exponential growth of women participating in competitive sports throughout the century. This was made possible through several initiatives. The International Olympic Committee (IOC) has played an important role in advocating for gender equity by encouraging women to participate in sports at all levels by creating more opportunities for female athletes and by expanding the Olympic program to include more women’s events [4]. The passage and implementation of Title IX as a federal law in the United States, allowing equal opportunity for athletic participation across sexes, have also significantly influenced the growth of women in sports at the collegiate and professional levels [2,5••].

Women now make up 43.7% of collegiate athletes across all division levels and 48.2% of NCAA division 1 power 5 conference athletes [5••]. In addition, there has been an increase in the number of women involved in intercollegiate sports as athletic administrators or part of the coaching and athletic training staff [6]. Unfortunately, this positive trend towards gender equity in sports has not yet reached women in medicine, especially in fields that care for elite athletes. Women comprise only 12.7% of all team physicians in the collegiate (18.1%) and professional (6.7%) levels, with the highest representation for female team physicians (31.3%) in the Women’s National Basketball Association (WNBA) [5••]. With this discrepancy in mind, it is important to consider how increased female team physician representation in sports might benefit our female athletes. We now have an expanded understanding of female athletes and how certain physiologic, biomechanical, and anatomic factors influence their risk for injury [7]. Similarly, we have an opportunity as clinicians to use this knowledge to optimize their health and performance [8,9••]. This paper will discuss specific areas that can be tailored to prevent injuries in female athletes and optimize their athletic performance.

## Tailoring Prevention to High-Risk Areas

### Growth and Developmental Considerations for Adolescent Female Athletes

Growth and development can be characterized by behavioral, physiological, and physical changes that are most pronounced during puberty. Girls and boys experience these changes differently [10]. Adolescent female athletes gain more body fat and less lean muscle mass than their male counterparts during puberty, which may increase female athletes’ risk for disordered eating, overtraining, and Relative Energy Deficiency in Sport [11]. Girls also tend to have greater ligamentous and joint laxity than boys that persists beyond puberty and can increase their risk for ligamentous injury such as ankle sprains [12]. Furthermore, while boys develop greater shoulder width and muscle mass, girls gain hip width and fat mass, which may contribute to their increased risk for ACL injuries. Prevention efforts should first start with increased awareness of these risks and increased focus on neuromuscular training for female athletes to mitigate these injury risks.

### Knee Injuries

Anterior cruciate ligament (ACL) tears are usually sustained from sports that involve cutting, pivoting, or twisting such as soccer, basketball, and football. Although relatively infrequent, ACL injuries can cause significant loss of time from play [13]. Female athletes are at increased risk of sustaining ACL tears from noncontact mechanisms compared to male athletes, especially after puberty. Female athletes also have a higher risk of subsequent contralateral ACL injury [9••]. Increased risk specific to female athletes may be attributed to multiple factors including anatomical (increased Q angle, narrow intercondylar notch, and increase in posterior tibial slope), hormonal, and neuromuscular (poor core and gluteal muscle strength and increased quadriceps-to-hamstring ratio) [9••,^13^]. Injury prevention programs that focus on biomechanical and neuromuscular factors such as competence in landing and cutting positions (e.g., box drop and landing) and hip and core strengthening (e.g., single-leg squat) can prevent these injuries, particularly when the prevention programs are initiated during pre-season and continued into the sports season as part of the athlete’s warmup exercises [8,9••,^13^].

Anterior knee pain from patellofemoral pain syndrome (PFPS) is an overuse injury that more commonly affects adolescent female athletes than their male counterparts [9••]. Similar factors that increase female athletes’ risk for ACL tears are associated with PFPS including anatomic variants (patella alta, trochlear dysplasia), static and/or dynamic malalignment (increased Q angle, valgus lower extremity alignment), and deficits with strength and flexibility at the pelvic, femoral, and knee regions resulting to poor neuromuscular control [9••,^13^]. Identifying these risk factors specific to girls and women should be utilized for developing strength and conditioning programs that can be helpful for injury prevention and rehabilitation [8,9••,^13^].

### Bone Stress Injuries

Bone stress injuries occur when repetitive load exceeds the capacity of the bone to repair itself. These injuries are usually sustained by endurance athletes or dancers and commonly involve weight-bearing bones of the lower extremities or pelvis. These injuries are more prevalent in females than in males and are due to risk factors including the following: extrinsic (exercise volume, type, or intensity), intrinsic (biomechanics, muscle strength, balance, and limb alignment), and/or medical/psychologic (poor nutrition, eating disorder/disordered eating (ED/DE), low energy availability (EA), menstrual dysfunction, low bone mineral density) [9••]. Screening for risk factors, especially those that are more prevalent in female athletes such as ED/DE that can lead to low EA and subsequent menstrual dysfunction and low bone mineral density, is important for injury prevention [9••]. Bone stress injuries that reveal underlying medical/psychological conditions should prompt coordination with a multidisciplinary team for evaluation and management [9••]. Progressive resistance training has also been found to improve bone mineral density thereby preventing bone stress injuries [8].

### Concussions

A concussion is a traumatic brain injury induced by biomechanical forces that result in clinical signs and symptoms that reflect a functional disturbance rather than structural injury [14]. Earlier studies have shown higher rates of concussions reported in female athletes compared to male athletes that played sports with the same rules [9••,^13^]. Females were also found to report more symptoms than males, have longer recovery times, and suffer worse outcomes [15–17]. These differences are hypothesized to be driven by multiple factors including deficits with neck strength or stability that increase susceptibility to concussive events, hormonal fluctuations that may influence pain response and impact recovery outcomes, and greater incidence of pre-existing conditions that are associated with prolonged recovery [16,18]. However, a recent prospective study found no overall difference in concussion recovery between male and female Division I athletes, while female Division II/III athletes had longer recovery. This suggests that modifiable extrinsic factors such as access to an athletic trainer or sports medicine specialist may also influence concussion outcomes [19••]. Injury prevention remains the greatest challenge in concussion management. Some injury reduction strategies include neck strengthening exercises, technique modifications (e.g., tackling and checking), enforcement of existing rules or game rule changes, and educational programs that focus on risk awareness and reporting of symptoms [13]. Although studies exploring the relationship between hormonal fluctuations with respect to the menstrual cycle and concussion recovery outcomes have been emerging in the recent years, more research is necessary to further understand how to apply these concepts in injury prevention and management of concussions specifically to female athletes.

### Sexual Violence in Sport

The term sexual violence when used in this paper includes both sexual harassment (any unwanted conduct of a sexual nature) and sexual abuse (any sexual conduct where consent is not given). All athletes are at risk for sexual violence with prevalence ranging widely and suspected to be underestimated. The risk seems to be highest for elite athletes, athletes in sports where there is early specialization, and where intensive talent identification occurs around puberty [20]. These factors all portend an environment of increased dependence on the coach and increased athlete vulnerability. Female athletes seem to be at increased risk for sexual violence in sport compared to male athletes, though the body of literature on this topic is heterogeneous with different definitions of sexual abuse and type of perpetrator and/or limited to very specific female athlete populations [21].

Due to the rising concern about sexual violence in sport, both the IOC and the American Medical Society for Sports Medicine (AMSSM) have recently published consensus statements focused on prevention and treatment of sexual violence in sport [22,23•]. With respect to actions recommended for sports medicine physicians and healthcare providers, the first task is raising awareness. Clinicians must be aware that sexual violence occurs in sport and must be committed to reducing the risk of sexual violence in sport as part of an overarching goal of fostering a culture of safety and health. Secondly, the team physician should collaborate with coaching and sports organizations on policies and procedures to prevent sexual violence in sport. Training should be provided to athletes, coaches, and administrators on the prevention and identification of sexual violence in sport [23•]. Policies should be developed that specify the context and extent of contact between the coach, healthcare providers, and the athletes [22,24]. Prudent recommendations include the following: never be alone in a room with an athlete or share a hotel room at an event, never drive an athlete home after practice, and avoid seeing an athlete socially [20]. Clinicians should have the knowledge and skills to recognize the signs and symptoms of sexual violence, evaluate and treat the athlete in a clinical setting, and report the allegation appropriately while adhering to patient confidentiality laws [23•].

## Optimizing Performance for Female Athletes

### How Does the Menstrual Cycle Affect Exercise Performance?

The menstrual cycle is made up of three phases of hormonal fluctuation that occur over the course of 23–28 days: the follicular phase (low estrogen, low progesterone levels), the ovulatory phase (high estrogen, low progesterone levels), and the mid-luteal phase (high estrogen and progesterone levels). Typically, we focus on the effects of estrogen and progesterone on the uterus in the setting of reproductive physiology; however, estrogen and progesterone also have effects on multiple organ systems, and these might have implications for female athletic performance.

Estrogen promotes muscle strength, whereas loss of estrogen leads to muscle weakness (as seen in menopause). Post-menopausal women who take estrogen hormone therapy had 5% greater strength than those women who did not take estrogen hormone therapy, a result of improved muscle function rather than muscle hypertrophy [25]. Estrogen and progesterone both affect the body’s metabolism of fat, protein, and carbohydrate; however, exactly how and if this affects athletic performance is not clear as the extent of metabolic demand caused by exercise also determines whether or not effects by estrogen and progesterone are significant [26].

Whether or not there are phases of the menstrual cycle that correlate with improved athletic performance remains debatable. Some studies have shown improved performance in each of the three phases of the menstrual cycle while others have shown no difference. McNulty et al. conducted the first meta-analysis to evaluate studies on exercise performance across the different phases of the menstrual cycle in eumenorrheic women. The authors found a very small effect size with reduced performance in the early follicular phase of the menstrual cycle when compared to the other phases. They concluded that this trivial reduction in athletic performance in the early follicular phase is so small that it is likely irrelevant for the majority of athletes but could be important for elite athletes where very small differences matter. They recommend that providers working with elite athletes be aware that performance might be reduced during the early follicular phase compared to all other phases. They also recommend that future studies compare exercise performance across multiple phases of the menstrual cycle, with objective measurements of blood hormone levels, to evaluate the effects of different ratios of estrogen to progesterone on performance [27].

### Does Use of Combined Estrogen-Progestin Oral Contraceptives Affect Exercise Performance?

Up to 57% of female college athletes take combined estrogen-progestin oral contraceptives (COCs) [28]. Benefits of COC use for the female athlete include high contraceptive efficacy, rapid reversibility, regulation of menstrual bleeding, decreased menstrual blood loss, improvement in iron deficiency anemia related to blood loss, decreased dysmenorrhea, reduced symptoms of premenstrual syndrome and premenstrual dysphoric disorder, and reduced risk of benign breast disease [29]. The effect of COCs on exercise performance is unclear based on existing data. In the first ever meta-analysis on the subject, Eliott-Sale et al. evaluated the effect of COCs on athletic performance and found that COCs might lead to a slight exercise performance deficit compared to naturally menstruating female athletes. However, the authors caution that the group-level effect is most likely trivial and that there was relatively large study design variance that might influence the effects. The authors recommend that clinicians take an individualized approach when counseling female athletes about the risks and benefits of COCs as the state of the existing data does not allow for more generalized guidance [30]. Because the negative effect of COCs on exercise performance is likely insignificant for the majority of athletes, this risk may in many athletes’ cases be outweighed by the numerous clear benefits of COCs.

### How Do Breast Biomechanics Affect Physical Activity?

Physical inactivity is a major public health problem in the United States. In 2017 only 49% of women in the United States achieved 150 min of moderate-intensity aerobic activity or 75 min of vigorous-intensity aerobic activity per week, while 28.1% of adult women reported engaging in no leisure-time physical activity [31]. Physical activity decreases in females as breast size increases [32,33]. Seventeen percent of women surveyed about breast health and physical activity reported that the breast was a barrier to physical activity, indicating that the inability to find the right sports bra and embarrassment about their breast motion were barriers to activity [34].

A correctly fitting bra can reduce exercise-related breast pain, avoid deep furrows due to strap pressure, and reduce the risk for neck and back pain [35]. Exercise-induced breast pain is thought to be the result of the breasts hitting the torso when a woman’s foot strikes the ground when she runs [36]. When women wear more supportive bras, they experience less breast motion and less exercise-induced breast pain. There are three main types of sports bras (crop top bra, encapsulation bra, and hybrid sports bra) [37•]**.** In general, crop top bras are less supportive than encapsulation bras. The best sports bra, however, is not necessarily the sports bra that maximally restricts breast motion, as sports bras that eliminate breast motion have been found to be uncomfortable. McGhee and Steele published a list of design features to consider when choosing a sports bra [37•]**.** The following are some key concepts from their publication that a sports medicine physician could discuss with patients when giving advice on bra fit: (1) The cups or front panel should completely cover each breast with no bulging of the breast over the top nor wrinkling or gapping of the fabric. The cups or front panel should have a side panel, sling, or side seams that reduce medial-lateral breast motion; (2) when wearing a new bra, the wearer should use the loosest hook so that as the bra stretches the wearer can fasten more securely as needed; (3) when the wearer is reaching overhead, the band of the bra should not slide up; (4) the bra straps should be wide and padded to support the breasts and adjusted so that they neither dig into nor slide off the shoulders; and (5) underwires do not add to support but have to do with breast shape. If the bra has an underwire, it should sit flat against the ribs and not on the breast tissue [37•]**.**

### How Can We Optimize Health and Athletic Performance in the Pregnant and Postpartum Athlete?

The American College of Obstetricians and Gynecologists encourages women to initiate or continue participating in physical activities during and after pregnancy due to its multiple health benefits. Although some modifications may be necessary due to anatomic and physiologic changes that occur during pregnancy, exercise is considered safe for women with uncomplicated pregnancies or those without medical contraindications. Both aerobic exercises (walking, stationary cycling, and dancing) and strength and conditioning exercises (stretching and resistance training) have been proven to be beneficial, while contact activities with high risk for abdominal trauma should be avoided. Scuba diving is contraindicated during pregnancy due to concerns surrounding the fetal cardiopulmonary circulation.

Limited data is available for elite athletes who participate in vigorous exercise during pregnancy. A recent meta-analysis on elite athletes and pregnancy outcomes suggests a decrease in pregnancy-related low back pain and an increased odds of excessive weight gain compared with active/sedentary controls. The same study found no significant difference in other pregnancy-related outcomes (e.g., miscarriage, preterm birth, low birth weight, macrosomia, cesarean section, instrumental delivery, perineal tears, and maternal mental health conditions). All studies included in this systematic review were observational and were evaluated as “very low” to “low” certainty of evidence [38•]**.** Competitive athletes who engage in these activities must have a clear understanding of the risks and require close supervision by healthcare professionals to ensure that they avoid hyperthermia, maintain proper hydration, and sustain adequate caloric intake necessary for fetal growth [39•]**.**

Maintenance of regular exercise is important in optimizing athletic performance for the pregnant and postpartum athlete. Multiple studies have shown that regular physical activity during pregnancy and the postnatal period, as compared to inactivity, reduces the risk of developing depression [40–42]. In addition, aerobic exercise for about 30–60 min two to seven times per week during pregnancy, as compared with being more sedentary, is associated with a significantly reduced risk of gestational hypertensive disorders overall and cesarean delivery [43].

A prospective study showed that pregnant women who continued to participate in sports that specifically trained the perineal muscles (dance, artistic gymnastics, rhythmic gymnastics, athletics, figure skating, tennis, volleyball, basketball, soccer, baseball, running, horseback riding, and snowboarding) are associated with a lower rate of episiotomy and ≥ 2nd-degree perineal lacerations [44]. Stress urinary incontinence has been reported in a large variety of sports and can interfere with training and compromise athletic performance [45]. Regular exercisers at mid-pregnancy have stronger pelvic floor muscles than their sedentary peers, and this pelvic floor muscle strength is associated with urinary continence [46].

Women who develop gestational diabetes or anemia during pregnancy are at risk for poor postpartum fitness [47]; these are modifiable risk factors that should be addressed with regular moderate-intensity physical activity and adequate iron supplementation during pregnancy in order to optimize athletic performance during the postpartum period. Besides low iron, magnesium deficiency may also reduce physical performance and negatively affect exercise capacity, as it plays a central role in the control of neuronal activity, cardiac excitability, neuromuscular transmission, muscular contraction, vasomotor tone, and blood pressure [48]. A loss of intracellular magnesium can lead to muscle weakness, neuromuscular dysfunction, and cramping or spasms. The recommended intake of magnesium increases during pregnancy and lactation. While adequate magnesium intake can help to optimize athletic performance both during and after pregnancy, the use of supplemental magnesium has not been shown to enhance athletic performance.

Strong evidence exists that moderate intensity physical activity in women during and following pregnancy has multiple health benefits and reduces the risk of pregnancy-related complications [49]. Presence of these medical conditions can certainly diminish or delay the return of peak athletic performance; thus, counseling pregnant women to continue exercising can be recommended to optimize health and athletic performance.

Pregnancy-related low back pain (LBP) is often accepted as a normal part of pregnancy, with a prevalence of 20 to 84%, and while it is often thought that back pain will resolve after delivery, it can continue postpartum in 50% of women at 1 year and 20% at 3 years [50]. Pelvic girdle pain (PGP) is a separate entity up to four times more prevalent and even more disabling. Few studies specifically address LBP and PGP in pregnant athletes, with inconsistent results regarding the influence of regular exercise on PGP and LBP during pregnancy [51]. Some studies have found that kinesiotape or other similar drug-free elastic therapeutic tapes have a short-term effect on both pain intensity and disability in women with pregnancy-related low back pain, although there was no sham taping application in the control groups, and therefore, there could have been a placebo effect [52]. However, with this type of tape being used by many athletes, physical therapists, and athletic trainers for the treatment of various musculoskeletal problems to help activate the muscle, reduce pain, reposition joints, and reduce abnormal muscular tension, it will likely become even more popular as a complementary and safe treatment method to achieve effective control of LPB and PGP in active pregnant women.

### How Does Strength and Conditioning Affect Exercise Performance?

Strength and conditioning (S&C) can both optimize athletic performance and decrease injury risk in athletes. Though research specific to female athletes in regard to S&C is limited, the research that exists demonstrates that girls and women stand to benefit significantly from strength training. A recent meta-analysis of a limited number of studies on resistance training (RT) in female athletes found that male and female youth athletes show similar RT-related gains in muscle strength and vertical jump performance, while girls had significantly larger training-induced sport-specific performance improvement [53]. Female athletes benefit the most from S&C programs incorporated before the onset of puberty as this timing most effectively builds muscle mass [54]. This is an important consideration because sex differences in performance are exaggerated during adolescence where girls typically reach a plateau or improve minimally. According to the National Strength and Conditioning Association’s (NSCA) most recent recommendations, plyometric programs should be instituted before puberty due to their ability to facilitate the already naturally occurring neural process during this time and add strength training in the post-pubertal age [55]. With respect to adult female athletes, strength training builds bone density, reduces risk for falls, improves glycemic control, reduces pain and stiffness related to osteoarthritis, and reduces risk of cardiovascular disease [56].

Despite the known benefits of S&C, S&C is an underutilized type of physical activity in the United States. The Centers for Disease Control and Prevention (CDC) recommend that children perform muscle-strengthening activities such as climbing or doing push-ups at least 3 days per week and adults perform strengthening activities at least 2 days per week [57]. For athletes, disparities exist in the incorporation of S&C programs in male vs female training regimens, with female athletes having less access to S&C programs [53,54]. This seems to be partly due to both personal barriers, such as females reporting fear of “bulking up” leading to less interest in S&C programs, as well as systemic barriers wherein females are not being challenged to work as hard as males because they are viewed as dainty. A study of S&C programs among varsity high school athletes found that only 17% of female athletes were required to participate in S&C year-round compared to 50% of male athletes. In addition, coaches of female athletes were less likely to know the credentials of their strength coaches and were less likely to use certified coaches to plan and implement their strength and conditioning programs [58].

## Optimizing Workforce to Care for Female Athletes

### Why Is Gender Equity Important in the Team Physician Workforce in Order to Optimize Care for Female Athletes?

Gender equity is important in medicine not only for ethical reasons, to give women equal opportunities professionally as those of men [59], but also in terms of quality of care. Diversity promotes excellence in all fields, medicine included [60]. In quality metrics, women outperform men in a number of areas. Women physicians are more likely than men physicians to screen their patients for breast and cervical cancer [61]. Women physicians engage in more patient-centered communication than men physicians [62]. Patients of women physicians had lower 30-day readmission and 30-day mortality rates post-hospitalization than did patients of men physicians [63••]**.** A Swedish study found that female general practitioners (GP), female surgeons, and older male physicians were least paternalistic, and among female physicians, the most autonomy-respecting groups were female surgeons and GPs [64].

When patients can better identify with their physician based on sex or ethnicity, patients demonstrate greater trust, better outcomes, and improved communication and compliance [62,65–68]. In some cases, women patients prefer women physicians. A 2018 survey of women patients showed that 43% of those surveyed preferred a woman endoscopist. Eighty-seven percent of those who preferred a woman endoscopist were willing to wait > 30 days for the procedure to be performed by a woman physician. Five percent of the women surveyed reported that they would not have a colonoscopy unless performed by a woman endoscopist [69], raising concern that care might be delayed if gender concordance were not met. In a quantitative and qualitative study of collegiate athletes, the majority of male and female respondents expressed no preference for gender of team physician; however, only 51.6% of women collegiate athletes reported that they were comfortable seeing a physician of the opposite sex for an examination of the reproductive organs or an issue involving sexual health versus 75.5% of males [70]. In another study of Division I collegiate athletes, women athletes preferred women physicians when the patients had questions about sex, contraception, acne, diet, relationships, and mental health [71]. Similar to the study on colonoscopy, one worries that if female athletes have a preference for a female physician when discussing these subjects, will they avoid seeking care if they do not have access to a woman physician?

In the sports medicine literature, it has been found that females drop out of sports participation more commonly than males [72–77]. Women suffering from ACL or other knee injuries were significantly less likely to return to competitive sport than men [78]. A qualitative study of varsity collegiate athletes returning to sport following surgery found that female athletes found difficulty within interpersonal relationships and external support, while male athletes struggled internally with their body image and changing self-concept. Being female, young, having a limited experience of injury, negative emotion, and perceptions of isolation are factors related to less successful outcomes of rehabilitation [79]. Female primary care sports medicine physicians and orthopedic sports medicine surgeons may be able to better understand the experiences of female athletes, critical to ensure that they are supported as they cope with injury and seek to successfully return to sport [80].

### What Is the Current State of the Sports Medicine Physician Workforce?

In the United States, sports medicine certification is available through the American Board of Medical Specialties to physicians with certification in the specialties of family medicine, internal medicine, pediatrics, emergency medicine, physical medicine and rehabilitation, and orthopedic surgery, who complete an Accreditation Council for Graduate Medical Education–accredited sports medicine fellowship. Sport medicine physicians, also referred to as primary care sports medicine (PCSM) physicians, treat patients with both medical and musculoskeletal conditions, while orthopedic sports medicine surgeons focus on the surgical and non-surgical management of musculoskeletal conditions.

Based on the 2019–2020 annual report of the AMSSM, females comprise 28% of total members (1154/4122) [81]. In the American Orthopaedic Society for Sports Medicine (AOSSM) membership database, female orthopedic surgeons comprised 6.5% of members (239/3668). Women are not only underrepresented in the field of sports medicine, but they are also less likely to become a team physician. Overall, men sports medicine-family physicians (SM-FP) are more likely to be team physicians than women SM-FPs (71.3% vs 45.3%), and of those SM-FPs who covered a professional team, only 7% were women [82]. In a survey of Division I colleges and professional sports teams, women comprised the minority of team physicians although they constituted a similar percentage of orthopedic surgeons to their representation in AOSSM (with some regional differences at the collegiate level) [5••]**.**

Data collected for NBA and WNBA team physicians between the years of 2009 and 2019 found that of the 125 NBA team physicians, 122 (97.6%) were male and 3 (2.4%) were female; of the 28 WNBA team physicians, 20 (71.4%) were male and 8 (28.6%) were female. The Northeast had the highest proportion of female team physicians, with 5 of 18 (27.8%); the lowest was in the West with 1 of 48 (2.1%) [83•]**.** While WNBA had the highest percentage of female team physicians among professional leagues, much lower proportions were found in the all-male professional leagues of the NBA (6.3%), NFL (1.9%), and MLB (7.9%) [5••]**.**

In 2019, data was collected on head team physician and athletic trainer composition within the NCAA. Out of the 1121 NCAA institutions included, only 11.2% (129/1145) of the head team physicians were women, while 88.7% were men. Data also revealed that 31.7% head athletic trainers were women, with significant gender differences among the 3 NCAA divisions. Within NCAA institutions, fewer female physicians and athletic trainers than men have sports medicine leadership roles [84].

### How Can We Improve the Gender Balance Within the Sports Medicine Workforce?

Barriers that women in sports medicine face are similar to those experienced by women in other fields of medicine and science. The 2019 data from the American Medical Colleges Association show that while women now comprise 50.5% of all medical school students, women remain underrepresented in upper faculty positions [85]. There also remains a gender gap in promotion and leadership in medicine that has remained unchanged in the last 35 years [86]. Women associate and full professors are still half as likely as men of equal rank to be appointed to department chair. These differences in promotion persist across every academic department. Because women are also underrepresented among residency program directors and on editorial boards of medical journals, they are less likely to serve as role models and help with research publications and career advancement for other women. A study comparing the ratio of male and female participants in original research articles published in Sports and Exercise Medicine journals found that female researchers were significantly under-represented (39% female vs 61% male). The average percentage of female participants per article was also low, ranging from 35 to 37% [87]. “Manels,” or panels at medical conferences or meetings consisting only of men, are unfortunately still common and convey implicit gender discrimination and bias [88]. Despite the higher quality care provided by women physicians, men continue to receive more pay, more promotion, and more leadership opportunities than women.

While motherhood and the inability to have work–life balance may inhibit some female sports medicine physicians from moving up to leadership positions in collegiate and other athletic settings, hopefully as more women assume leadership positions such as the head team physician, more younger women will experience mentorship and seek out these positions as well. However, challenges for women in sports medicine leadership include those common to women in general: [89]

1. The association of leadership with masculine qualities;
2. Lack of affirmation of feminine leadership traits and the resulting lack of self-identification as a leader;
3. Barriers in the career progression pipeline;
4. Lack of diversity in selection panels;
5. The “double bind” (where women are expected to demonstrate masculine traits and are then criticized for doing so);
6. Persisting cultural norms (career vs family).

Potential causes of the sex gap in promotion to leadership positions include a persisting “old boys’ club” mentality and climate, lack of sex parity in leadership and compensation, and a disproportionate burden of family responsibilities resulting in difficulties in achieving work–life balance. While female and male sports medicine physicians early in their careers may have had similar leadership aspirations, women were less likely to perceive their institution as willing to make changes to address diversity goals and especially to be family friendly.

Thorborg et al. discussed that gender balance and gender diversity are needed in order to promote a move towards gender justice within sports medicine [90]. Female sports medicine physicians may need to challenge those — often male physicians — who are considered the experts and hold the leadership positions. Female sports medicine physicians may need to advocate to expand the number of topics relevant to women athletes at conferences and the number of publications authored by women in sports medicine journals. It is important to address the “manels” by asking for transparency and involvement in the invitation process.

In summary, women comprise a minority of team physicians in select NCAA Division I collegiate and especially professional sports organizations. There is also a substantial difference in the number of female physicians with leadership roles in both the NBA and WNBA compared to male physicians. Barriers affecting female sports medicine physicians and sports medicine orthopedic surgeons as team physicians should be identified and strategies implemented to provide equal opportunities to both male and female physicians. Lastly, because the current gender composition of SM-FPs is not representative of the current gender composition of family medicine residents, future studies should examine the motivation and barriers for women pursuing SM training and careers.

## Conclusions

There has been an exponential growth in women participating in competitive sports throughout the years. Team physicians must understand the physiologic, biomechanical, and anatomic factors that are unique to female athletes in order to tailor injury prevention programs and optimize their athletic performance. Despite the achievements made by women in sports, women physicians are still underrepresented in fields that care for elite athletes. Advocating for gender equity in sports medicine by identifying barriers and implementing strategies to advance representation of women in the field will increase workforce diversity and promote excellence in sports medicine care.

## References

[1] International Olympic Committee. 2019. Key dates in the history of women in the Olympic Movement. *Int Olympic Comm*. [cited 2020 Dec 1] Available from: https://www.olympic.org/women-in-sport/background/key-dates.

[2] Bell R. Academy USS. A history of women in sport prior to title IX. *Sport J*. 2008;22:1543–9518. https://thesportjournal.org/article/a-history-of-women-in-sport-prior-to-title-ix/.

[3] Gregg EA, Gregg VH (2017). Women in sport: historical perspectives. Clin Sports Med..

[4] International Olympic Committee. 2019. Promotion of women in sports through time. International Olympic 741 Committee. [cited 2020 Dec 1] Available from: https://www.olympic.org/women-in-sport/background. Accessed December 1, 2020.

[5] O’Reilly OC, Day MA, Cates WT, Baron JE, Glass NA, Westermann RW (2020). Female team physician representation in professional and collegiate athletics. Am J Sports Med..

[6] Acosta, R.V., & Carpenter LJ (2014). *Women in intercollegiate sport: a longitudinal national study thirty seven year update 1977-2014*. 2014:1–56.

[7] Statuta SM, Wood CL, Rollins LK (2020). Common medical concerns of the female athlete. Prim Care Clin Off Pract..

[8] Hilibrand MJ, Hammoud S, Bishop M, Woods D, Fredrick RW, Dodson CC (2015). Common injuries and ailments of the female athlete; pathophysiology, treatment and prevention. Phys Sportsmed..

[9] •• Female athlete issues for the team physician: a consensus statement - 2017 Update. Curr Sports Med Rep. 2018; 10.1249/JSR.0000000000000482. **Identifies sports injuries with special focus on female athletes and emphasis on preventive efforts** 10.1249/JSR.0000000000000482 29738322

[10] Hurvitz M, Weiss R. The young female athlete. Pediatr Endocrinol Rev. 2009;7(2):43–9. 10.1016/s0278-5919(20)30213-1 20118893

[11] IndridadoIr, M. H., Sveinsson, T., Magnusson, K. T., Arngrimsson, S. A., & Johannsson, E. Algengi íróttameidsla, íróttaátttaka og broMall vegna meidsla hjá 17 og 23 ára ungmennum. Laeknabladid, 2015;101(10):451–456. 10.17992/lbl.2015.10.45 10.17992/lbl.2015.10.45 26444230

[12] Corso M (2018). Developmental changes in the youth athlete: implications for movement, skills acquisition, performance and injuries. J Can Chiropr Assoc..

[13] Gregory A, Kerr Z, Parsons J, Panel E (2016). Selected issues in injury and illness prevention and the team physician: a consensus statement. Curr Sports Med Rep..

[14] McCrory P, Meeuwisse W, Dvořák J (2017). Consensus statement on concussion in sport—the 5th international conference on concussion in sport held in Berlin, October 2016. Br J Sports Med..

[15] Covassin T, Elbin RJ, Harris W, Parker T, Kontos A (2012). The role of age and sex in symptoms, neurocognitive performance, and postural stability in athletes after concussion. Am J Sports Med..

[16] Mollayeva T, El-Khechen-Richandi G, Colantonio A (2018). Sex & gender considerations in concussion research. Concussion..

[17] Koerte IK, Schultz V, Sydnor VJ, Howell DR, Guenette JP, Dennis E, Kochsiek J, Kaufmann D, Sollmann N, Mondello S, Shenton ME, Lin AP (2020). Sex-related differences in the effects of sports-related concussion: a review. J Neuroimaging..

[18] Wunderle K, Hoeger KM, Wasserman E, Bazarian JJ (2014). Menstrual phase as predictor of outcome after mild traumatic brain injury in women. J Head Trauma Rehabil..

[19] Master CL, Katz BP, Arbogast KB (2020). Differences in sport-related concussion for female and male athletes in comparable collegiate sports: a study from the NCAA-DoD Concussion Assessment, Research and Education (CARE) Consortium. Br J Sports Med..

[20] Marks S, Mountjoy M, Marcus M (2012). Sexual harassment and abuse in sport: the role of the team doctor. Br J Sports Med..

[21] Parent S, Lavoie F, Thibodeau MÈ, Hébert M, Blais M (2016). Sexual violence experienced in the sport context by a representative sample of Quebec adolescents. J Interpers Violence..

[22] Mountjoy M, Brackenridge C, Arrington M, Blauwet C, Carska-Sheppard A, Fasting K, Kirby S, Leahy T, Marks S, Martin K, Starr K, Tiivas A, Budgett R (2016). International Olympic Committee consensus statement: harassment and abuse (non-accidental violence) in sport. Br J Sports Med..

[23] Koontz JS (2020). This article has been co-published in the. Br J Sports Med..

[24] International Olympic Committee. Olympic movement medical code in force as from 31 March 2016. [cited 14 Dec 2020] Available from: https://stillmed.olympic.org/media/Document%20Library/OlympicOrg/IOC/Who-We-Are/Commissions/Medical-and-Scientific-Commission/Olympic-Movement-Medical-Code-31-03-2016.pdf. 2016;(March):1-11.

[25] Lowe, D. A., Baltgalvis, K. A., & Greising, S. M. Mechanisms behind estrogen’s beneficial effect on muscle strength in females. Exercise and Sport Sciences Reviews, 2010;38(2):61–67. 10.1097/JES.0b013e3181d496bc. 10.1097/JES.0b013e3181d496bc PMC2873087 20335737

[26] Oosthuyse T, Bosch AN. *The effect of the menstrual cycle on exercise metabolism implications for exercise performance in eumenorrhoeic women*. Sports Medicine. 10.2165/11317090-000000000-00000 10.2165/11317090-000000000-00000 20199120

[27] McNulty KL, Elliott-Sale KJ, Dolan E (2020). The effects of menstrual cycle phase on exercise performance in eumenorrheic women: a systematic review and meta-analysis. Sport Med..

[28] Verrilli LE, Landry M, Blanchard H (2017). Contraceptive choices and menstrual patterns in high level female athletes. Fertil Steril..

[29] Allen, R.H. Combined estrogen-progestin oral contraceptives: patient selection, counseling, and use. In: UpToDate, Post TW (Ed), UpToDate, Waltham, MA. (Accessed on December 30, 2020).

[30] Elliott-Sale KJ, McNulty KL, Ansdell P (2020). The effects of oral contraceptives on exercise performance in women: a systematic review and meta-analysis. Sport Med..

[31] Centers for Disease Control and Prevention Division of Nutrition, Physical Activity and Obesity. Nutrition, physical activity, and obesity: Data, trends and maps. [cited 2020 Dec 30] Available from: https://nccd.cdc.gov/dnpao_dtm/rdPage.aspx?rdReport=DNPAO_DTM.ExploreByLocation&rdRequestForwarding=Form. Accessed December 30, 2020.

[32] Coltman CE, Steele JR, McGhee DE (2019). Does breast size affect how women participate in physical activity?. J Sci Med Sport..

[33] Spencer L, Fary R, McKenna L, Jacques A, Lalor J, Briffa K (2020). The relationship between breast size and aspects of health and psychological wellbeing in mature-aged women. Women’s Heal..

[34] Burnett E, White J, Scurr J (2015). The influence of the breast on physical activity participation in females. J Phys Act Heal..

[35] McGhee DE, Steele JR (2010). Optimising breast support in female patients through correct bra fit. A cross-sectional study. J Sci Med Sport..

[36] Mcghee DE, Steele JR (2020). Breast biomechanics: what do we really know?. Physiology (Bethesda)..

[37] McGhee DE, Steele JR (2020). Biomechanics of breast support for active women. Exerc Sport Sci Rev..

[38] Wowdzia JB, McHugh TL, Thornton J, Sivak A, Mottola MF, Davenport MH (2021). Elite Athletes and pregnancy outcomes: a systematic review and meta-analysis. Med Sci Sports Exerc..

[39] • Physical activity and exercise during pregnancy and the postpartum period: ACOG Committee Opinion, Number 804. Obstet Gynecol. 2020;135:e178–88. 10.1097/AOG.0000000000003772**Updates in exercise recommendations for pregnant women**. 10.1097/AOG.0000000000003772 32217980

[40] Nakamura A, van der Waerden J, Melchior M, Bolze C, El-Khoury F, Pryor L (2019). Physical activity during pregnancy and postpartum depression: systematic review and meta-analysis. J Affect Disord..

[41] Kołomańska-Bogucka D, Mazur-Bialy AI (2019). Physical activity and the occurrence of postnatal depression—a systematic review. Med..

[42] Chang C, Putukian M, Aerni G (2020). Mental health issues and psychological factors in athletes: detection, management, effect on performance and prevention: American Medical Society for Sports Medicine Position Statement—Executive Summary. Br J Sports Med..

[43] Magro-Malosso ER, Saccone G, Di Tommaso M, Roman A, Berghella V (2017). Exercise during pregnancy and risk of gestational hypertensive disorders: a systematic review and meta-analysis. Acta Obstet Gynecol Scand..

[44] Uccella S, Manzoni P, Marconi N, Toscani C, Biasoli S, Cianci S, Franchi M, Sorice P, Bertoli F, Zorzato PC, Gallina D, Ghezzi F, Serati M (2019). Impact of sport activity and physical exercise on obstetrical and perineal outcomes at delivery: A prospective study. Am J Perinatol..

[45] de Mattos Lourenco TR, Matsuoka PK, Baracat EC, Haddad JM (2018). Urinary incontinence in female athletes: a systematic review. Int Urogynecol J..

[46] Bø K, Ellstrøm Engh M, Hilde G (2018). Regular exercisers have stronger pelvic floor muscles than nonregular exercisers at midpregnancy. Am J Obstet Gynecol..

[47] Miller MJ, Kutcher J, Adams KL (2017). Effect of pregnancy on performance of a standardized physical fitness test. Mil Med..

[48] Bohl CH, Volpe SL (2002). Magnesium and exercise. Crit Rev Food Sci Nutr..

[49] Dipietro L, Evenson KR, Bloodgood B, Sprow K, Troiano RP, Piercy KL, Vaux-Bjerke A, Powell KE, 2018 PHYSICAL ACTIVITY GUIDELINES ADVISORY COMMITTEE* (2019). Benefits of physical activity during pregnancy and postpartum: an umbrella review. Med Sci Sports Exerc..

[50] Noon ML, Hoch AZ (2012). Challenges of the pregnant athlete and low back pain. Curr Sports Med Rep..

[51] Haakstad LAH, Bø K (2011). Effect of regular exercise on prevention of excessive weight gain in pregnancy: a randomised controlled trial. Eur J Contracept Reprod Heal Care..

[52] Kaplan Ş, Alpayci M, Karaman E (2016). Short-term effects of kinesio taping in women with pregnancy-related low back pain: a randomized controlled clinical trial. Med Sci Monit..

[53] Zebis MK, Andersen LL, Brandt M, Myklebust G, Bencke J, Lauridsen HB, Bandholm T, Thorborg K, Hölmich P, Aagaard P (2016). Effects of evidence-based prevention training on neuromuscular and biomechanical risk factors for ACL injury in adolescent female athletes: a randomised controlled trial. Br J Sports Med..

[54] Sommi C, Gill F, Trojan JD, Mulcahey MK (2018). Strength and conditioning in adolescent female athletes. Phys Sportsmed..

[55] Lloyd RS, Cronin JB, Faigenbaum AD, Haff GG, Howard R, Kraemer WJ, Micheli LJ, Myer GD, Oliver JL (2016). National strength and conditioning association position statement on long-term athletic development. J Strength Cond Res..

[56] Seguin, R. A., Epping, J. N., Burchner, D. M., Bloch, R., & Nelson, M. E. *Growing Stronger: strength training for older adults*. Medicine & Science in Sports & Exercise, 2009;41(Supplement 1):51.

[57] Physical activity recommendations for different age groups | Physical Activity | DNPAO | CDC. https://www.cdc.gov/physicalactivity/basics/age-chart.html. Accessed January 10, 2021.

[58] Reynolds ML, Ransdell LB, Lucas SM, Petlichkoff LM, Gao Y (2012). An examination of current practices and gender differences in strength and conditioning in a sample of varsity high school athletic programs. J Strength Cond Res..

[59] American Medical Women’s Association. Gender Equity Task Force. [cited 2020 Dec 30] Available from: https://www.amwa-doc.org/our-work/initiatives/gender-equity-task-force/. Accessed December 30, 2020.

[60] Association of American Medical Colleges. 2020. AAMC Statement on Gender Equity. [cited 2020 Jan 30] Available from: https://www.aamc.org/what-we-do/diversity-inclusion/aamc-statement-gender-equity.

[61] Franks P, Bertakis KD. Physician gender, patient gender, and primary care. J Womens Health (Larchmt). 2003 Jan-Feb;12(1):73-80. 10.1089/154099903321154167 10.1089/154099903321154167 12639371

[62] Roter DL, Hall JA (2004). Physician gender and patient-centered communication: a critical review of empirical research. Annu Rev Public Health..

[63] Tsugawa Y, Jena AB, Figueroa JF, Orav EJ, Blumenthal DM, Jha AK (2017). Comparison of hospital mortality and readmission rates for medicare patients treated by male vs female physicians. JAMA Intern Med..

[64] Lynöe N, Juth N, Helgesson G (2010). How to reveal disguised paternalism. Med Heal Care Philos..

[65] Thom DH, Hall MA, Pawlson LG (2004). Measuring patients’ trust in physicians when assessing quality of care. Health Aff..

[66] Shen MJ, Peterson EB, Costas-Muñiz R (2018). The effects of race and racial concordance on patient-physician communication: a systematic review of the literature. J Racial Ethn Heal Disparities..

[67] Alsan M, Garrick O, Graziani G (2019). Does diversity matter for health? Experimental Evidence from Oakland. Am Econ Rev..

[68] Monzani D, Vergani L, Pizzoli SFM (2020). Sexism interacts with patient–physician gender concordance in influencing patient control preferences: findings from a vignette experimental design. Appl Psychol Heal Well-Being..

[69] Menees SB, Inadomi JM, Korsnes S, Elta GH. Women patients' preference for women physicians is a barrier to colon cancer screening. Gastrointest Endosc. 2005;62(2):219–23. 10.1016/s0016-5107(05)00540-7. 10.1016/s0016-5107(05)00540-7 16046982

[70] Wesner ML, Vallance J. Athletes' preference for gender of team physician. Clin J Sport Med. 2007;17(2):143–4. 10.1097/JSM.0b013e31802b4fa6. 10.1097/JSM.0b013e31802b4fa6 17414484

[71] Holschen JC, Singal BM (2006). College athletes’ preference of physician gender. Clin J Sport Med..

[72] Fokkema T, Hartgens F, Kluitenberg B (2019). Reasons and predictors of discontinuation of running after a running program for novice runners. J Sci Med Sport..

[73] Indriðadóttir MH, Sveinsson Þ, Magnússon KÞ, Arngrímsson SÁ, Jóhannsson E (2015). Algengi íþróttameiðsla, íþróttaþátttaka og brottfall vegna meiðsla hjá 17 og 23 ára ungmennum. Læknablaðið..

[74] Deelen I, Ettema D, Kamphuis CBM (2018). Time-use and environmental determinants of dropout from organized youth football and tennis. BMC Public Health..

[75] da Silva DRP, Werneck AO, Collings P (2019). Identifying children who are susceptible to dropping out from physical activity and sport: a cross-sectional study. Sao Paulo Med J..

[76] Howie EK, Ng L, Beales D, McVeigh JA, Straker LM (2019). Early life factors are associated with trajectories of consistent organized sport participation over childhood and adolescence: Longitudinal analysis from the Raine Study. J Sci Med Sport..

[77] Eime R, Harvey J, Charity M (2018). Girls’ transition from participation in a modified sport program to club sport competition - a study of longitudinal patterns and correlates. BMC Public Health..

[78] Ardern CL, Webster KE, Taylor NF, Feller JA (2011). Return to the preinjury level of competitive sport after anterior cruciate ligament reconstruction surgery. Am J Sports Med..

[79] Forsdyke D, Smith A, Jones M, Gledhill A (2016). Psychosocial factors associated with outcomes of sports injury rehabilitation in competitive athletes: a mixed studies systematic review. Br J Sports Med..

[80] Morgan AM, Fernandez CE, Terry MA, Tjong V (2020). A qualitative assessment of return to sport in collegiate athletes: does gender matter?. Cureus..

[81] American Medical Society for Sports Medicine. 2019-2020 Annual Report of the American Medical Society for Sports Medicine (AMSSM). [cited 2020 Dec 30] Available from: https://www.amssm.org/Content/pdffiles/2019-20AnnualReport.pdf. Accessed December 30, 2020.

[82] Cox R, Morgan ZJ, Nithyanandam S, Puffer JC, Peterson LE (2020). Practice patterns of family physicians with and without sports medicine certification. Clin J Sport Med..

[83] Hinkle AJ, Brown SM, Mulcahey MK (2020). Gender disparity among NBA and WNBA team physicians. Phys Sportsmed..

[84] Lewis C, Jin Y, Day C (2020). Distribution of men and women among NCAA head team physicians, head athletic trainers, and assistant athletic trainers. JAMA Intern Med..

[85] Association of American Medical Colleges. 2019. The majority of U.S. medical students are women, new data show. [cited 2020 Dec 30] Available from: https://www.aamc.org/news-insights/press-releases/majority-us-medical-students-are-women-new-data-show. Accessed December 30, 2020.

[86] Richter KP, Clark L, Wick JA, Cruvinel E, Durham D, Shaw P, Shih GH, Befort CA, Simari RD (2020). Women physicians and promotion in academic medicine. N Engl J Med..

[87] Costello JT, Bieuzen F, Bleakley CM (2014). Where are all the female participants in Sports and Exercise Medicine research?. Eur J Sport Sci..

[88] Bekker S, Ahmed OH, Bakare U (2018). We need to talk about manels: the problem of implicit gender bias in sport and exercise medicine. Br J Sports Med..

[89] Tulloh L (2019). They call us fellows: the challenge of gender bias in the Australasian College of Sport and Exercise Physicians. Br J Sports Med..

[90] Thorborg K, Krohn L, Bandholm T (2020). ‘More Walk and Less Talk’: Changing gender bias in sports medicine. Br J Sports Med..

